# Evaluating the Acceptability, Swallowability, and Palatability of Film-Coated Mini-Tablet Formulation in Young Children: Results from an Open-Label, Single-Dose, Cross-Over Study

**DOI:** 10.3390/pharmaceutics15061729

**Published:** 2023-06-14

**Authors:** Juliane Münch, Isabelle Sessler, Hans Martin Bosse, Manfred Wargenau, Janine D. Dreesen, Giulio Loforese, Nicholas J. A. Webb, Rama Sivasubramanian, Sibylle Reidemeister, Philipp Lustenberger, Viviane Klingmann

**Affiliations:** 1 Department of General Pediatrics, Neonatology and Pediatric Cardiology, University Children’s Hospital Düsseldorf, Medical Faculty, Heinrich-Heine-University, 40225 Düsseldorf, Germany; juliane.muench@med.uni-duesseldorf.de (J.M.); isabelle.sessler@med.uni-duesseldorf.de (I.S.); hansmartin.bosse@med.uni-duesseldorf.de (H.M.B.); 2 M.A.R.C.O. GmbH & Co. KG, Institute for Clinical Research and Statistics, 40211 Düsseldorf, Germany; manfred.wargenau@marco-institut.de (M.W.); janine.dreesen@marco-institut.de (J.D.D.); 3 Global Drug Development, Novartis Pharma AG, 4002 Basel, Switzerland; giulio.loforese@novartis.com (G.L.); nicholas.webb@novartis.com (N.J.A.W.); sibylle.reidemeister@novartis.com (S.R.); philipp.lustenberger@novartis.com (P.L.); 4 Global Drug Development, Novartis Healthcare Pvt. Ltd., Hyderabad 500081, India; rama.sivasubramanian@novartis.com

**Keywords:** acceptability, mini-tablets, palatability, pediatric formulations, swallowability

## Abstract

Mini-tablets are advantageous over liquid formulations in overcoming challenges related to stability, taste, and dosage. This open-label, single-dose, cross-over study investigated the acceptability and safety of drug-free, film-coated mini-tablets in children aged 1 month–6 years (stratified: 4–6 years, 2–<4 years, 1–<2 years, 6–<12 months, and 1–<6 months), and their preference for swallowing either a high quantity of 2.0 mm or a low quantity of 2.5 mm diameter mini-tablets. The primary endpoint was acceptability derived from swallowability. The secondary endpoints were investigator-observed palatability, acceptability as a composite endpoint derived from both swallowability and palatability, and safety. Of 320 children randomized, 319 completed the study. Across all tablet sizes, quantities and age groups, acceptability rates based on swallowability were high (at least 87%). Palatability was rated as “pleasant/neutral” in 96.6% of children. The acceptability rates as per the composite endpoint were at least 77% and 86% for the 2.0 mm and 2.5 mm film-coated mini-tablets, respectively. No adverse events or deaths were reported. Recruitment in the 1–<6-months group was stopped early due to coughing—evaluated as “choked on” in three children. Both 2.0 mm and 2.5 mm film-coated mini-tablets are suitable formulations for young children.

## 1. Introduction

Designing pharmaceutical dosage formulations for children remains a major challenge due to the unique needs and characteristics of this patient population [1]. Evidence shows that over 60% of drugs are being used as off-label or off-license in children due to unavailability of child-appropriate dosage and formulations for many diseases [2,3]. The lack of trials in children and thus the lack of evidence for treatments in this population may result in the administration of potentially inadequate doses [4]. This may lead to an increased risk of potentially insufficient treatment or adverse reactions, including death, and deprives children of the full benefit of therapeutic advances. Liquid formulations provide dosing flexibility and ease of swallowing. However, they suffer from challenges of stability and sometimes require special storage conditions which may not be feasible in certain geographies of the world due to lack of facilities. In addition, they may have undesirable taste, which result in inadequate, inconsistent, or highly variable dosing [5]. Notably, liquid formulations such as suspensions are known to result in surprisingly unreliable dosing, with substantial underdosage or overdosage in children [6]. Thus, it is not only necessary to investigate the efficacy and optimal doses of pharmaceutical substances in different pediatric age groups but also to develop adapted galenic formulations for the most suitable routes of administration. In clinical practice, the specific pediatric requirements for adequate dosing depend on the age and physical development stage of the child, but the major deficiencies involve the availability of the required strength of formulation, the child’s ability to ingest standard-size solid dosage formulations, and the taste of oral medicines.

European Medical Agency (EMA) Guideline on pharmaceutical development of medicines for pediatric use, acknowledge that “different oral formulations may be age-appropriate but the acceptability of the size and shape of the tablets by target age group(s) should be justified, and where relevant supported by appropriate studies or clinical evidence. It should be noted that limited data are available in the literature regarding the influence of size, shape and the number of tablets on acceptability in different pediatric age groups” [7].

Therefore, there is a need for scientifically sound data to compare various oral pediatric administration routes, considering the suitability and capability of children, particularly at a young age, to ingest different galenic formulations. This can improve the safety and reliability of drug administration. In pediatric practice, syrup is the most commonly used formulation, but specially designed mini-tablets have advantages as they are easy to handle and have more stability, lower amount of potentially toxic excipients, and easier storage conditions [5]. Previous studies have demonstrated the suitability of mini-tablets for children of different age groups, including newborns, with superiority over liquid formulations in terms of swallowability and palatability. Thomson et al., demonstrated the suitability of 3 mm diameter mini-tablets in 2 to 6-year-old children [8], while a pilot study showed that administration of a little smaller mini-tablet of 2 mm diameter were suitable in younger children aged 6 months to <6 years [9]. Additional studies demonstrated the suitability of the uncoated and coated 2 mm mini-tablet in children between 6 months to <6 years [10], and the suitability of the uncoated mini-tablet in newborns [11]. Results showed that it was even superior to the syrup in most of the investigated age groups. Taking these results into account, the revised version of the EMA Guideline on pharmaceutical development of medicines for pediatric use from 2014 no longer has any age recommendation for solid oral dosage forms anymore [7]. Following this, a clinical trial that was performed in infants (aged 6 to 23 months) and toddlers (aged 2 to 5 years) showed that the administration of 25, 100, and 400 uncoated 2 mm mini-tablets in comparison to syrup was well accepted [12]. Moreover, larger tablets (oblong-tablets, 2.5 × 6 mm) have already been investigated and showed very good acceptability, swallowability, and palatability in children aged 1 to 5 years [13].

However, there is still lack of scientifically sound data on the suitability of larger mini-tablets for children of different age groups, particularly on coated mini-tablets. Therefore, it is important to conduct a clinical trial in a sufficiently large number of participants of the particularly vulnerable age groups, namely 1 month to 6 years, comparing the acceptability, swallowability and palatability of the more common 2.0 mm and the less common 2.5 mm film-coated mini-tablets in children.

The principal aim of this study was to assess the acceptability, swallowability, and palatability of multiple drug-free film-coated mini-tablets in children aged 1 month to 6 years. The study also sought to compare whether children of different age groups preferred a high quantity of smaller (2.0 mm) or a low quantity of larger (2.5 mm) mini-tablets. Data obtained from this study could be helpful in selecting an optimal mini-tablet size for pediatric patients.

## 2. Materials and Methods

### 2.1. Objectives

The primary objective of the study was to investigate the acceptability of a high quantity of 2.0 mm diameter film-coated mini-tablets compared to a low quantity of 2.5 mm diameter film-coated mini-tablets in children aged from 1 month to 6 years, accounting for the requirement of weight-based dosing in different age groups. The final dose for the active drug iptacopan in children of <12 years of age is still subject to ongoing pediatric investigations, and for the purpose of this acceptability study a realistic high-dose scenario was chosen leading to the administration of the indicated number of drug-free mini-tablets.

The secondary objectives were (i) to compare the swallowability of 2.0 mm and 2.5 mm film-coated mini-tablets in children aged from 1 month to 6 years; (ii) to compare the palatability of 2.0 mm and 2.5 mm film-coated mini-tablets in children aged from 1 month to 6 years; (iii) to identify any possible problem that could occur during swallowing; (iv) to identify the percentage of children who inhaled or coughed during ingestion of any of the two sizes of the oral formulations; and (v) to investigate the safety of the film-coated, drug-free mini-tablets.

### 2.2. Formulations

**Figure 1:**
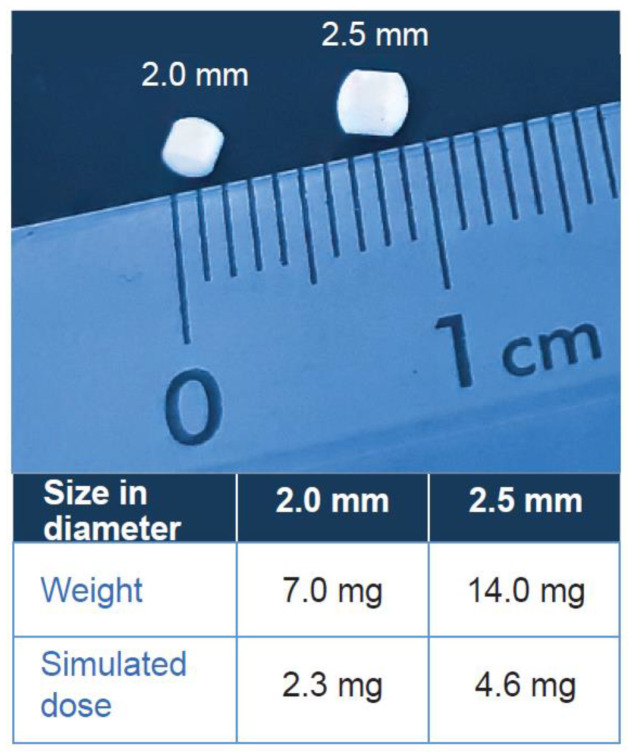
Drug-free mini-tablets. Simulated dose denotes the estimated amount of active drug that could be potentially administered in each mini-tablet size.

The mini-tablets were either 2.0 mm or 2.5 mm diameter in size, with a sphere-like shape, and were manufactured by Novartis (Figure 1). The increase in tablet diameter by 0.5 mm increases the volume of the tablet such that the drug dose can be doubled while retaining a relatively small tablet size. Both mini-tablet formulations are drug-free, consisting of microcrystalline cellulose, lactose monohydrate, colloidal anhydrous silica, croscarmellose sodium, and magnesium stearate in the core and a coating layer containing sodium lauryl sulfate, stearic acid, basic butylated methacrylate copolymer, and talc. The coating material used for the prototypes tested dissolves at pH up to 5 and protects the mini-tablets from dissolving rapidly in the oral cavity (normal pH range 6.2–7.6) which impacts acceptability and palatability [14,15].

### 2.3. Study Participants

The study was registered in the Deutsches Register Klinischer Studien (DRKS, German Clinical Trial Register) no. DRKS00027631. Male and female children aged 1 month to 6 years, who were inpatients or outpatients (for any reason) at the Department of General Pediatrics, Neonatology and Pediatric Cardiology of the University Hospital Düsseldorf, Germany, between 31 January 2022, and 25 April 2022, were recruited for this study. Children were stratified into five age groups (Group 1: 4–6 years, Group 2: 2–<4 years, Group 3: 1–<2 years, Group 4: 6–<12 months, and Group 5: 1–<6 months). The inclusion and exclusion criteria of the study participants are shown in Table 1.

### 2.4. Study Design

**Figure 2:**
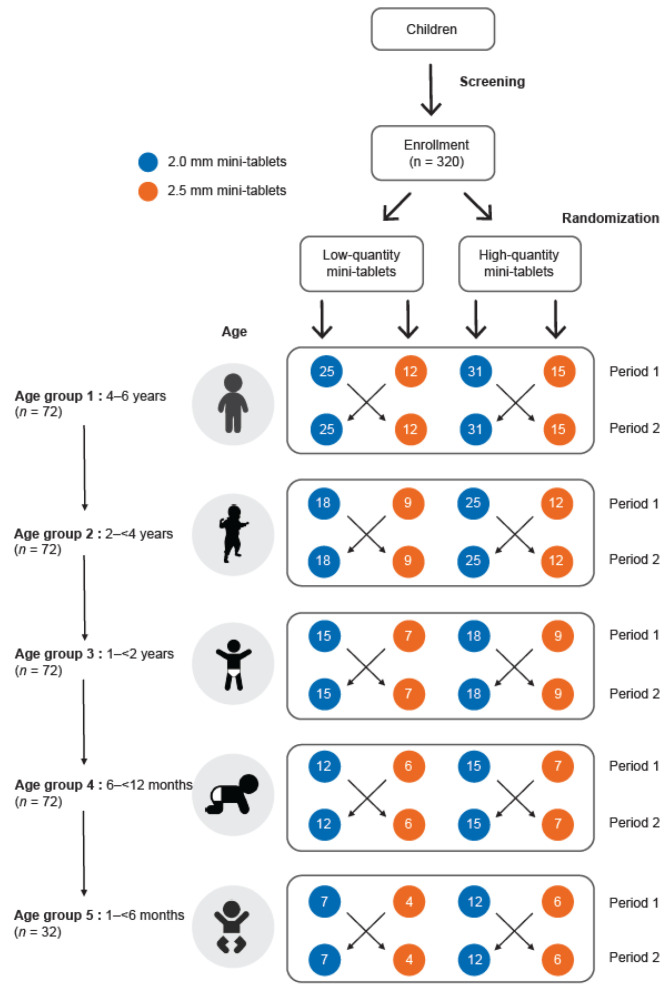
Study design.

The study design is shown in Figure 2. This open-label, randomized, cross-over study was conducted in five different age groups (Group 1: 4–6 years, Group 2: 2–<4 years, Group 3: 1–<2 years, Group 4: 6–<12 months, and Group 5: 1–<6 months). Participants were recruited in a descending order starting with the oldest age group (i.e., 4–6 years). Once one-third of the required participants in this group had been successfully investigated without any safety concerns or adverse events, recruitment in the next younger age group (Group 2: 2–<4 years) began in parallel. The remaining recruitment was carried out concurrently with sentinel recruitment of the consecutive younger age groups, following the same procedure as for the previous age groups.

Each group was randomized to two parallel arms to receive either a low or a high quantity of drug-free, film-coated, mini-tablets. The two quantities (high and low) for both mini-tablet sizes were tested in order to compare and evaluate the number of tablets that will be needed to cover the doses required for the age range that is being tested as the doses are calculated based on body weight. Children in each arm received the film-coated mini-tablets in two consecutive periods, Period 1, and Period 2. Within each arm, the 2.0 mm and 2.5 mm film-coated mini-tablet sub-groups were compared in a randomized cross-over fashion. The precise number of mini-tablets in the low- or high-quantity mini-tablet arm was determined by the child’s age, respectively (4–6 years: 25 vs. 12 or 31 vs. 15; 2–<4 years: 18 vs. 9 or 25 vs. 12; 1–<2 years: 15 vs. 7 or 18 vs. 9; 6–<12: 12 vs. 6 or 15 vs. 7, and 1–<6 months: 7 vs. 4 or 12 vs. 6). Variations in the number of tablets within the low or high quantity mini-tablet arms, allows an estimation of the expected dosage range for different age groups based on the World Health Organization (WHO) growth chart [16].

#### 2.4.1. Randomization

Randomization was performed by stratified age groups and dosing arms using a self-developed and validated SAS macro RANDOM, which is based on the SAS function RANUNI for uniformly distributed variables. Children were randomized into two arms—low or high quantity mini-tablets. In a second step, children were assigned to one of the two treatment sequences (sequence 1 = 2.5 mm mini-tablets in Period 1 cross-over to 2.0 mm mini-tablets in Period 2; and sequence 2 = 2.0 mm mini-tablets in Period 1 cross-over to 2.5 mm mini-tablets in Period 2; Figure 2).

#### 2.4.2. Administration Procedure

During the examination phase, the child and their parent(s) or legal guardian, were seated in a quiet area. Instructions were given to children in a standardized manner using age-appropriate language. The 2.0 mm or the 2.5 mm film-coated mini-tablets were given with either soft food (yoghurt or fruit mousse) or drinks (water, tea, milk, or juice) according to the choice of the child, and their parent(s) or legal guardians. For soft food, mini-tablets were placed on a teaspoon with food and swallowed together. For drinks, the child swallowed the film-coated mini-tablets with a mouthful of drink administered immediately after the mini-tablets.

The trained investigator observed the swallowing process and the child’s reaction (e.g., swallowed, chewed/left over, spat out, choked on, or refused to take). About 45 s after placing the film-coated mini-tablets into the child’s mouth, the mouth was inspected by the investigator to ensure proper ingestion of the mini-tablet. Palatability was assessed by a second trained investigator, who observed the immediate physical reactions of the child after placing the formulation in child’s mouth.

The procedures were repeated in Period 2 with the other formulation.

### 2.5. Endpoints

The primary endpoint was defined as the acceptability derived from swallowability as assessed by five scoring criteria from swallowability, Table 2. The secondary endpoints included palatability (as a binary outcome: ‘pleasant’ or ‘other’) and acceptability derived from the composite acceptability endpoint of swallowability and palatability and expressed as binary outcome (‘yes’, high or good acceptability or ‘no’, low or no acceptability). Adverse events (AEs) were also monitored and reported.

### 2.6. Evaluation Criteria

Swallowability and palatability were assessed separately, and their scoring criteria are provided in Table 2. The scoring criteria for assessing acceptability derived from a composite endpoint of swallowability and palatability, is also shown in Table 2. Acceptability as a composite endpoint was defined as “high”, “good”, “low”, or “no” based on function of swallowability and the rating based on investigator’s observation of palatability.

### 2.7. Determination of Sample Size

For each cross-over cohort (i.e., for each dosing arm in each age group), a sample size of 36 participants (18 per sequence group) was regarded as appropriate to address the primary objective. Based on this sample size, the 90% confidence interval (CI) for an anticipated difference of at least 15% points (e.g., 90% vs. 75%) between treatment acceptability rates would not have included zero. Furthermore, pooling of the data from the two cross-over designs for each age group (if justifiable) would lead to a sample size of 72 participants for each age group for the comparison of the 2.0 mm and the 2.5 mm tablet regimens. Based on 72 participants, a difference of at least 15% points (e.g., 90% vs. 75%) could have been detected with 80% power at a significance level α = 10% (two-sided). As a result, a total of 360 participants were planned to be randomized.

### 2.8. Statistical Analyses

All statistical calculations were carried out using SAS language and procedures (SAS 9.4 version, SAS-Institute, Cary, NC, USA). The imputation of missing data was not performed. The evaluation variables, endpoints, and safety data were summarized descriptively by treatment regimens. Categorical data were summarized using frequencies and percentages, and continuous data by number of observations, mean, standard deviation, minimum, first quartile, median, third quartile, and maximum.

The primary endpoint of acceptability was analyzed as binary outcome according to the cross-over design. Acceptability rates were compared between the two treatment regimens “high quantity of coated mini-tablets of 2.0 mm” and “low quantity of coated mini-tablets of 2.5 mm diameter” by applying the analysis method proposed by Schouten and Kester [17]. At first, the difference in acceptability rates between the two regimens was estimated for each sequence group and then averaged over both sequence groups in a second step. This analysis was performed for each cross-over setting in each age group, and for the combined cross-overs for each age group. Overall rates were analyzed by comparing the pooled data of low and high quantities of 2.0 mm tablets and low and high quantities of 2.5 mm tablets across all age groups.

The secondary endpoints were analyzed accordingly. The endpoint analyses were performed on the full-analysis-set which however was identical to the safety analysis set (participants who had received at least one administration of film-coated mini-tablets). This particularly means that major violations of any inclusion or exclusion criterion did not occur and that all participants had at least one post-enrolment endpoint assessment.

## 3. Results

### 3.1. Disposition of Participants

**Figure 3:**
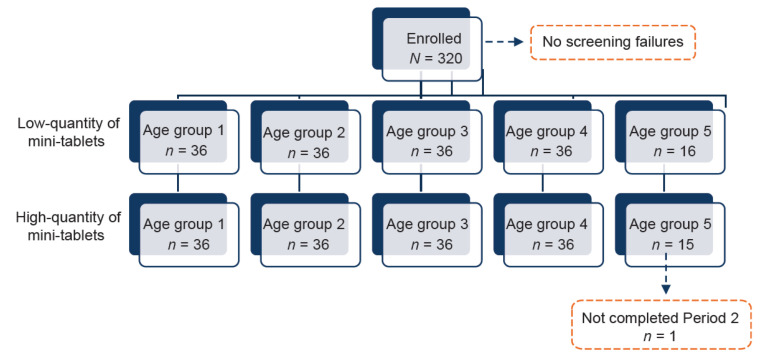
Disposition of participants. Recruitment of children <6 months of age was prematurely stopped as decided by the investigator owing to “choked on” event in three children.

A total of 320 children across five age groups (stratified: 4–6 years, 2–<4 years, 1–<2 years, 6–<12 months, and 1–<6 months) were included in the study, with 72 participants in each age group and 36 per age group in each dosing arm (2.0 mm 2.5 mm mini-tablets), except age group 5; Figure 3. One child in group 5 was withdrawn after the first administration due to coughing and thus a total of 319 children completed the study.

### 3.2. Primary Endpoint

**Figure 4:**
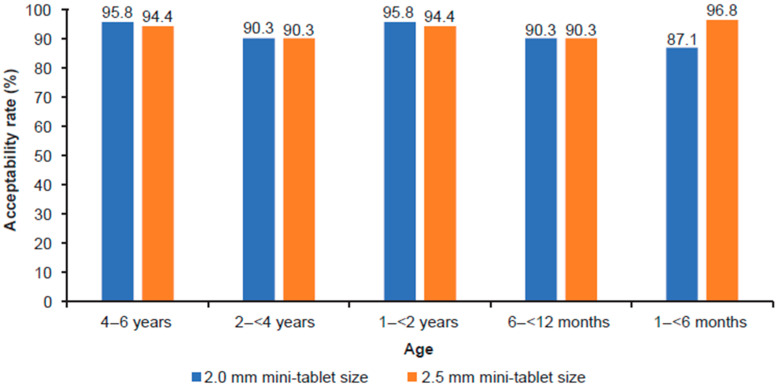
Acceptability rates based on swallowability criteria by age groups and tablet sizes. *n* = 36 per age group in each dosing arm, except 1–<6 months group, where *n* = 15 in 2.0 mm and *n* = 16 in 2.5 mm dosing arm.

The primary endpoint, acceptability rates based on swallowability, was high and comparable across tablet sizes, quantities, and age groups. The overall averaged difference between the two tablet sizes (2.0 mm minus 2.5 mm) was −0.3%, 90% CI −2.0 to 1.4; *p* = 0.757). The lowest acceptability rate was 87.1% in the youngest age group (which was not completed) for the 2.0 mm film-coated mini-tablets (Figure 4).

#### 3.2.1. Swallowability

**Figure 5:**
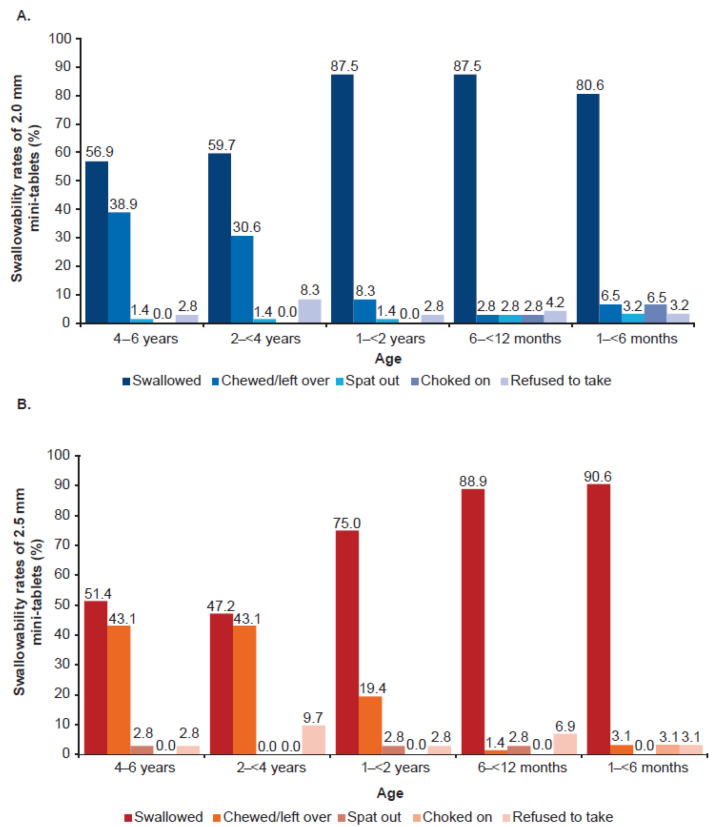
Swallowability rates by age group and (**A**) 2.0 mm and (**B**) 2.5 mm mini-tablet sizes. *n* = 36 per age group in each dosing arm, except 1–<6 months group, where *n* = 15 in 2.0 mm and *n* = 16 in 2.5 mm dosing arm.

Swallowability of the film-coated mini-tablets was assessed in children aged 1 month to 6 years based on five criteria (Table 2). The majority of children in all age groups were able to swallow both 2.0 mm and 2.5 mm mini-tablets, with a higher swallowability rate observed in the younger age groups (1–<2 years, 6–<12 months, and 1–<6 months). In comparison, the proportion of children who chewed/leftover the mini-tablets were lower across younger age groups (1–<2 years, 6–<12 months, and 1–<6 months). The proportion of children who spat out, choked on, or refused to take the tablets were very low with both 2.0 mm and 2.5 mm mini-tablets. There were two children in the age group 6–<12 months (both cases were reported in the 2.0 mm mini-tablets), and three children in the youngest (1–<6 months) age group (two cases were reported with the 2.0 mm mini-tablets and one was with the 2.5 mm mini-tablets) who coughed during study drug administration and were assessed as “choked on”, leading to discontinuation of recruitment in the youngest age group (Figure 5A,B).

### 3.3. Secondary Endpoints

#### 3.3.1. Palatability

**Figure 6:**
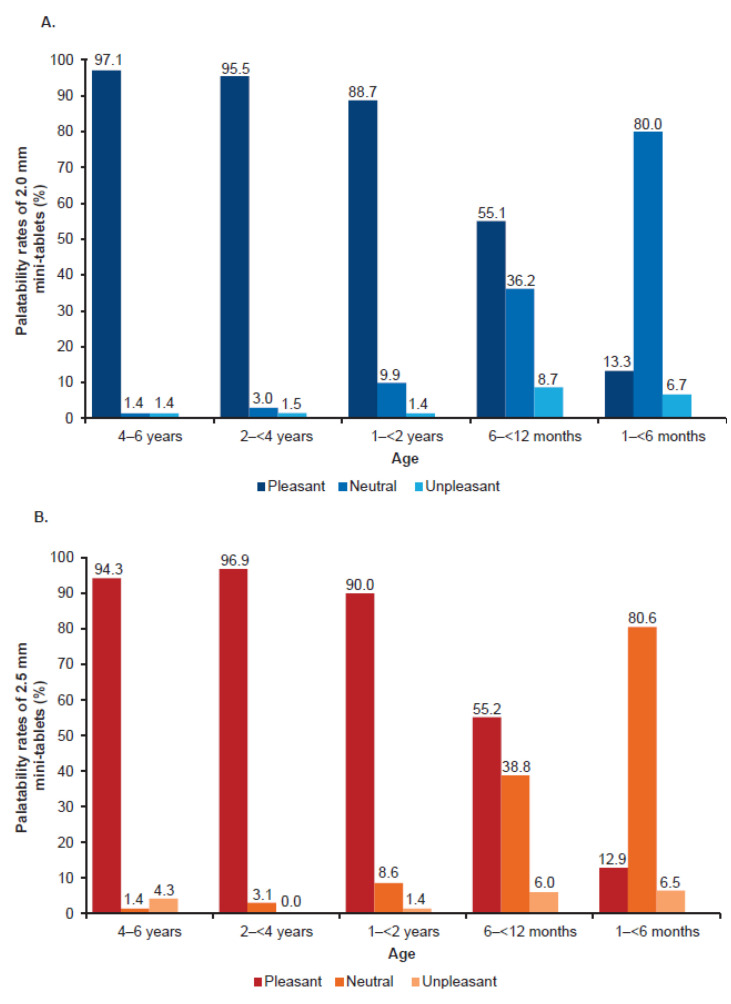
Palatability response based on child’s physical reactions by age groups and (**A**) 2.0 mm and (**B**) 2.5 mm mini-tablet sizes. *n* = 36 per age group in each dosing arm, except 1–<6 months group, where *n* = 15 in 2.0 mm and *n* = 16 in 2.5 mm dosing arm.

The palatability of the mini-tablets was generally rated as “pleasant” in the majority of children in age groups 1–<2 years, 2–<4 years, and 4–6 years, whereas palatability in the younger age groups (6–<12 months, and 1–<6 months) had mostly neutral ratings. An unpleasant reaction to the administration of both tablet sizes was found in less than 10% of the children in all age groups (Figure 6A,B).

Palatability was also evaluated as a binary endpoint, with the outcomes of “pleasant or neutral” versus “unpleasant” being considered. As a result, palatability was observed as pleasant/neutral in 93.5% up to 100% of the children, whereas an unpleasant palatability was assessed only in very few children of all age groups. Palatability (observed as pleasant/neutral) did not differ across all tablet sizes, quantities, and age groups. The overall averaged difference between the two tablet sizes (2.0 mm minus 2.5 mm) was 0.3%, 90% CI −0.9 to 1.6; *p* = 0.648.

#### 3.3.2. Acceptability as Composite Endpoint

**Figure 7:**
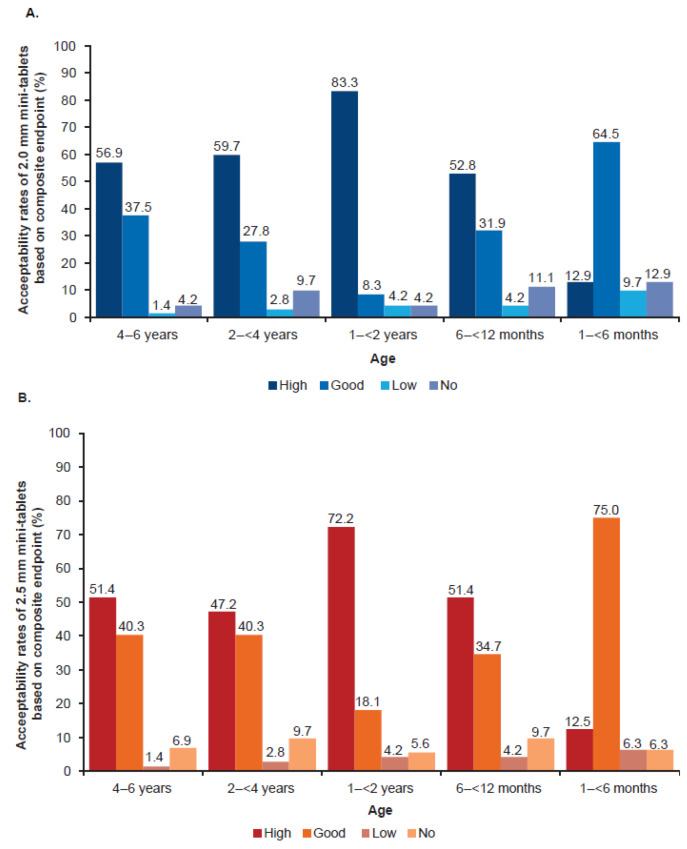
Acceptability rates as composite endpoint of swallowability and palatability by age group and (**A**) 2.0 mm and (**B**) 2.5 mm mini-tablet sizes. *n* = 36 per age group in each dosing arm, except 1–<6 months group, where *n* = 15 in 2.0 mm and *n* = 16 in 2.5 mm dosing arm.

The acceptability rates based on the composite endpoint of swallowability and palatability were comparable between the 2.0 mm and the 2.5 mm film-coated mini-tablets. High acceptability was most frequently observed in the age group 1–<2 years with 83.3% (2.0 mm) and 72.2% (2.5 mm) of the children, whereas these rates were very low in the age group 1–<6 months with 12.9% (2.0 mm) and 12.5% (2.5 mm). The youngest age group showed the highest proportion for good acceptability among both tablet sizes. Frequencies for acceptability as a composite endpoint in each age group are presented in Figure 7A,B.

**Figure 8:**
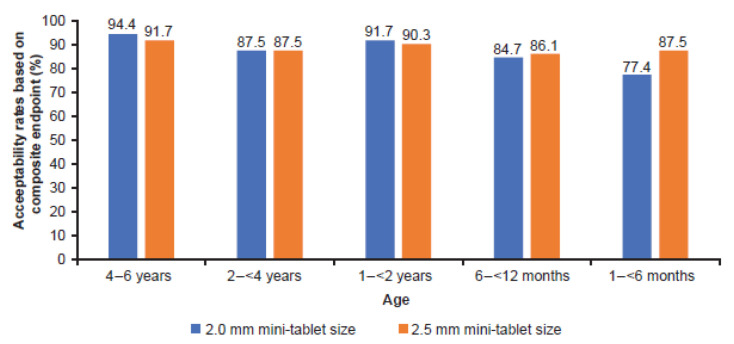
Acceptability determined by the composite endpoint of swallowability and palatability, derived from high and good acceptability results. *n* = 36 per age group in each dosing arm, except 1–<6 months group, where *n* = 15 in 2.0 mm and *n* = 16 in 2.5 mm dosing arm.

The acceptability rates based on high and good acceptability determined by the composite endpoint did not differ across all tablet sizes, quantities, and age groups. The overall averaged difference between the two tablet sizes (2.0 mm minus 2.5 mm) was −0.6%, 90% CI −2.6 to 1.3; *p* = 0.588; Figure 8.

#### 3.3.3. Safety Assessments

Coughing, which met the swallowability criterion “choked on”, was reported for two children in the age group 6–<12 months, and for three children in the age group 1–<6 months. None of these events were of clinical relevance and were not assessed as AEs. Nonetheless, as a precaution for infants in the age group 1–<6 months, the study was terminated due to clinical safety concerns. No AEs or deaths were reported in this study.

## 4. Discussion

This cross-over study provides evidence that the acceptability, swallowability, and palatability of a high quantity of smaller (2.0 mm) and a lower quantity of larger (2.5 mm) film-coated mini-tablets were similar in children of different age groups. The similarity in the acceptability of both sizes of mini-tablets indicates that increasing the tablet size by 0.5 mm does not reduce the acceptability. The larger mini-tablet size allows for doubling the dose of the active ingredient, thus enabling the possibility of formulating mini-tablets for a specific dosage, including high-dose therapies.

The study had a high power and credibility with a total of 320 children in two arms, and 72 participants in each age group, except the youngest group (*n* = 32), ensuring the validity of the results. Moreover, the cross-over study design allowed for the reduction of variability of data and the number of children required in this study.

The EMA Guideline on pharmaceutical development of medicines for paediatric use [7] and the Reflection paper on formulations of choice for the pediatric population [18] recommend various factors such as route of administration, dosage form, dosing flexibility, and excipients in the preparation when investigating acceptability of pediatric formulations. Moreover, acceptability is fundamental to effective pharmacotherapy and is defined as the ability and willingness of a patient to use and its caregiver to administer the medicine as intended [7]. Findings from our study showed a high rate of overall acceptability for mini-tablet formulations across all age groups, including children as young as 6 months up to 6 years. These are in line with previous research on acceptability of pediatric formulations [9,10,11,12,13].

Swallowing is a complex physiological process that requires the coordination of several muscles and nerves and occurs in multiple phases [19]. In young children, development of this process may vary from birth through adulthood [19]. Therefore, the main concern with solid oral formulations, especially in younger children, is their ability to swallow the formulation, which can increase the risk of inhalation and aspiration, which in turn could affect the ability to take solid dosage forms. Interestingly in this study, only a very few children in the age groups 1–<2 years, 6–<12 months, 1–<6 months had left-over mini-tablets in their mouths. In the current study, brief coughing—evaluated as “choked on” was experienced by two children in 6–<12 months (both for 2.0 mm mini-tablets) age group and three children (one for 2.5 mm and two for 2.0 mm mini-tablets) in the youngest age group (1–<6 months) when taking the film-coated mini-tablets. Film-coated mini-tablets do not dissolve immediately in the mouth and may increase the risk of aspiration. Although these data were reviewed by the Study Monitoring Board who did not raise any safety concerns, further recruitment of children in youngest age group was stopped due to investigators’ reservations. No AEs or deaths were reported in the study.

There were no cases of aspiration observed in this study, which is consistent with the previous studies that tested swallowability of uncoated mini-tablets in about 1000 children [9,10,11,12,13]. Although a few children aged 1–<6 months showed poor swallowing, the acceptability of mini-tablets was still high (87.1%) within this age group. These results are comparable with those from the earlier studies in neonates, which showed that acceptability was higher than swallowability [11].

The current study showed that a greater percentage of the older children (1–6 years) had chewed/left over tablets compared to the younger children (1–12 months). The ability of older children to chew and swallow solid foods is relatively well developed compared to younger children, and they may have a better control of their oral movements. This is also influenced by their prior experience with oral medications [20]. Thus, when asked to swallow the tablets, these children may inspect and assess the texture and probably chew it.

Another challenge with oral pediatric formulations is taste masking especially for liquid formulations [5]. Coating the solid oral pediatric formulations is a well-established method for taste masking. Palatability is an important criterion in pediatric age groups, since several drugs have a bitter taste and taste masking is necessary to enhance palatability, especially if the drug needs to be used for a prolonged period. In this study, both 2.0 mm and 2.5 mm film-coated mini-tablets were mostly assessed as pleasant across all age groups, except the youngest age group (1–<6 months) where most showed neutral reaction. Palatability assessed as neutral is sufficient for medications, as they are not intended to be enjoyed and this can help to discourage overdose of medications and misuse. The lack of positive experience of palatability in the youngest age group may be due to the differences in the developmental milestones in infants [21].

Acceptability rate based on swallowability alone was higher than the acceptability rate based on the composite endpoint of swallowability and palatability across all groups (97% vs. 88%, and 97% vs. 89%) for 2.0 mm and 2.5 mm, respectively), because palatability was assessed as “unpleasant” in a few children (ranging between one and six children per age group for the 2.0 mm film-coated mini-tablet, and between one and four children for the 2.5 mm film-coated mini-tablet). These results show that the new composite acceptability endpoint discriminates better in the assessment of acceptability than the calculation of acceptability based on swallowability alone.

The study has few limitations. One of the main limitations is that this was a single-dose cross-over study conducted in a hospital environment, and thus may lack generalizability of the results to a larger population or administration of the treatment for an extended period of time. Another limitation is that hospital-based studies are performed in a controlled environment, and thus the results may differ if the same treatment is applied in a home environment. Additionally, recruitment in the youngest age group (1–<6 months) was stopped earlier, so the results should be interpreted with caution for this age group.

The results from our pediatric study using the solid oral mini-tablets are encouraging and we believe this approach could be extended to other age groups. Eventually, study findings can be potentially implemented into and beyond the iptacopan pediatric program to provide more effective and convenient treatments for patients in all age groups. Importantly, this study was conducted as a part of a commitment to Health Authorities to develop an age-appropriate formulation for iptacopan (LNP023).

Iptacopan is an oral, first-in-class, highly potent, specific inhibitor of factor B of the alternative complement pathway [22], currently in development for the treatment of complement-mediated diseases (such as, complement 3 glomerulopathy [NCT05222412, NCT04817618], IgA nephropathy [NCT04557462], atypical hemolytic uremic syndrome [NCT05795140, NCT04889430], immune-complex membranoproliferative glomerulonephritis [NCT05755386], and paroxysmal nocturnal hemoglobinuria [NCT04747613, NCT04820530, NCT05630001]) [23,24,25]. Clinical studies are planned to evaluate the efficacy and safety of iptacopan for several indications in children and adolescents, using various pediatric formulations.

## 5. Conclusions

This single-dose, cross-over study demonstrated high acceptability of both sizes of film-coated mini-tablets (2.0 mm and 2.5 mm) for most children in all age groups, irrespective of the low or high quantity of tablets administered. The high acceptability rates were consistently observed throughout the study groups, regardless of the age group or tablet size with overall palatability being pleasant or neutral for over 90% of the children. These findings suggest that film-coated mini-tablets may be an excellent option for oral administration in young children between 6 months and 6 years. Importantly, our study suggests that additional monitoring is required to ensure safety in children aged 1–<6 months.

Overall, this study showed that oral film-coated mini-tablets are a promising option of pediatric formulation, and an alternative to the liquid formulations, which are likely to be unstable and challenging to store. Further research in other age groups would provide additional evidence for the use of film-coated mini-tablets.

## Figures and Tables

**Table 1:** Eligibility criteria.

Inclusion Criteria	Exclusion Criteria
Children aged ≥ 1 month to ≤6 years	Impairment of swallowing solids due to illness
Male and female	Lactose-intolerance
Ability to swallow	Premedication and concomitant medication that cause nausea, fatigue, or palsy
Willingness and capability of participants and participants’ parents or legal guardians to comply with examination procedures	Children in the post-operative period who were yet to commence regular oral feeding
Participant and/or participant’s parents or legal gaurdian capabable of providing written informed consent and assent where possible	Children, who had eaten one hour before examination and who afterwards feel sick because of the food

**Table 2:** Evaluation criteria.

**A. Scoring Criteria for Swallowability**			
**Score**	**Observation**		
**1**	**Swallowed**	No chewing took place during deglutition and no residuals of the solid were found during oral inspection.	
**2**	**Chewed/left over**	Chewing was observed before deglutition and/or that the whole or parts of the solid were found in the mouth during oral inspection and/or some mini-tablets left over on the spoon (≥80% should have been swallowed).	
**3**	**Spat out**	No deglutition took place and the solid was no longer in the child’s mouth.	
**4**	**Choked on**	The solid was swallowed the wrong way or a cough was caused.	
**5**	**Refused to take**	The child did not allow the investigator to place the solid in the mouth and/or <80% of mini-tablets swallowed.	
**B. Definition of acceptability derived from swallowability alone**			
	**Criteria**		
**Acceptable**	**Yes**	Swallowability score was 1 or 2	
	**No**	Swallowability score was >2 (3–5)	
**C. Scoring criteria for palatability**			
**Score**	**Assessment**	Interpretation	
**1**	**Pleasant**	Positive hedonic pattern: Tongue protrusion, smack of mouth and lips, finger sucking, corner elevation	
**2**	**Neutral**	Neutral mouth and body movements, and face expressions (irregular and involving lips)	
**3**	**Unpleasant**	Negative aversive pattern: Gape, nose wrinkle, eye squinch, frown, grimace, head shake, arm flail	
**D. Scoring criteria for composite acceptability endpoint derived from palatability and swallowability**			
**Palatability**	**Swallowability score**		
	**1**	**2**	**≥3**
**Pleasant**	High	Good	No
**Neutral**	Good	Low	No
**Unpleasant**	Low	No	No
**E. Acceptability as composite acceptability endpoint**			
**Yes:** High/good			
**No:** low/no			

## Data Availability

The data underlying this article cannot be shared publicly due to the privacy of individuals that participated in the study. The data will be shared on reasonable request to the corresponding author.
